# ISSR-Based Genetic Diversity Assessment of Genus *Jasminum* L. (Oleaceae) from Pakistan

**DOI:** 10.3390/plants10071270

**Published:** 2021-06-22

**Authors:** Naeem Akhtar, Ishfaq Ahmad Hafiz, Muhammad Qasim Hayat, Daniel Potter, Nadeem Akhtar Abbasi, Umer Habib, Adil Hussain, Hina Hafeez, Muhammad Ajmal Bashir, Saad Imran Malik

**Affiliations:** 1 Department of Horticulture, Pir Mehr Ali Shah Arid Agriculture University, Rawalpindi 46000, Pakistan; drishfaq@uaar.edu.pk (I.A.H.); nadeem.abbasi@uaar.edu.pk (N.A.A.); umer_habib@hotmail.com (U.H.); 2 Department of Plant Sciences, College of Agricultural and Environmental Sciences, University of California, Davis, CA 95616, USA; d.potter@ucdavis.edu; 3 Plant Systematics and Evolution Laboratory, Department of Plant Biotechnology, Atta ur Rahman School of Applied Biosciences (ASAB), National University of Sciences and Technology (NUST), Islamabad 44000, Pakistan; mqasimhayat@hotmail.com; 4 Department of Biotechnology, Faculty of Life Sciences, University of Okara, Okara 56130, Pakistan; aadil.iiu07@gmail.com; 5 National Institute for Genomics and Advanced Biotechnology, National Agriculture Research Center, Islamabad 44000, Pakistan; hinahafeez001@gmail.com; 6 Department of Agriculture and Forestry Sciences, University of Tuscia, San Camillo de Lellis, snc, 01100 Viterbo, Italy; 7 Department of Plant Breeding and Genetics, Pir Mehr Ali Shah Arid Agriculture University, Rawalpindi 46000, Pakistan; Saad.malik@uaar.edu.pk

**Keywords:** *Jasminum* L., genetic diversity, ISSR, Pakistan, polymorphism

## Abstract

The genus *Jasminum* L., of the family Oleaceae, includes many species occurring in the wild, or cultivated worldwide. A preliminary investigation based on inter-simple sequence repeats (ISSR) was performed to assess the genetic diversity among 28 accessions, representing nine species of *Jasminum* from various regions, representing a range of altitudes in Pakistan. A total of 21 ISSR primers were used, which produced 570 amplified bands of different sizes, with a mean polymorphic band percentage of 98.26%. The maximum resolving power, polymorphism information content, and index values of the ISSR markers recorded for primers 6, 16, and 19 were 0.40, 12.32, and 24.21, respectively. Based on the data of the ISSR markers, the resulting UPGMA dendrogram with the Jaccard coefficient divided the 28 accessions into two main clades. At the species level, the highest values for Shannon’s information index, polymorphism percentage, effective allele number, Nei’s genetic variations, and genetic unbiased diversity were found in *Jasminum sambac* L. and *J. humile* L., while the lowest were observed in *J. mesnyi* Hance and *J. nitidum* Skan. Based on Nei’s unbiased genetic identity pairwise population matrix, the maximum identity (0.804) was observed between *J. elongatum* Willd and *J. multiflorum* (Burm. f.) Andrews, and the lowest (0.566) between *J. nitidum* Skan. and *J. azoricum* L. Molecular variance analysis displayed a genetic variation of 79% among the nine populations. The study was aimed to established genetic diversity in *Jasminum* species using ISSR markers. With the help of this technique, we were able to establish immense intra- and interspecific diversity across the *Jasminum* species.

## 1. Introduction

The Oleaceae family comprises 28 genera with ~900 species, wherein the genus *Jasminum* L. alone comprises ~200 species [1]. Jasmine species grow in the wild in most of the world’s tropical areas, but especially in South East Asia. Southwestern and Southeast Asia are at the center of jasmine diversity, while a few wild species have also been documented in Africa, Asia, Australia, the Pacific Islands, and Europe. These species are considered to have been first cultivated during the Old World era, and in the New World, they are found as wild species [2]. Since the earliest times, several species of jasmine have been used for ornamental purposes due to their elegant star-shaped flowers and sweet scent. Globally, many species are cultivated for their great industrial, ornamental, and cultural characteristics. Jasmines are genetically diverse. They can be evergreen or deciduous, erect or climbing shrubs, with creamy, pink, yellow, or white non-scented or scented flowers. Some important species of jasmine are present in different regions of Pakistan, specifically the Himalayan, sub-Himalayan, and Salt Range areas representing the subtropical, temperate, and tropical areas of the country.

Among the *Jasminum* species, *J. grandiflorum* is found mostly in temperate to subtropical areas of the Salt Range of Pakistan [3]. Numerous variants of *J. humile* are widespread in the temperate and subtropical regions, from the upper parts of Kohistan to Abbottabad, Waziristan, and some temperate regions of Baluchistan. *Jasminum* species, such as *J. multiflorum* and *J. elongatum,* are found in the Murree hills, the temperate regions, and Nakial (Azad Jammu and Kashmir), the subtropical region of Pakistan. *J. leptophyllum* is a taxonomically described as a new endemic species, found in a relatively unexplored and remote part of Palas Valley, Kohistan [4,5]. Another *Jasminum* species, *J. mesnyi*, is commonly dispersed in the Margalla hills of Islamabad. The species *J. sambac* and *J. nitidum* and their cultivars are present and commonly cultivated all over Pakistan. Some species, such as *J.* *azoricum* and *J. polyanthum*, are rarely grown in Pakistan.

The majority of *Jasminum* species are found in the form of their synonyms, with almost 90 species considered diverse and real [6]. However, according to some researchers, *Jasminum* comprises only 64 diverse or real species [7]. The reason behind the synonymy of *Jasminum* species is the large number of accessions from different geographical regions of the world. With continuous variation in morphological characteristics, clear infraspecific classification becomes a problem [8]. This puzzling synonymy is also found in some prominent *Jasminum* species, such as *J. elongatum,* which is a variable and widespread tropical species across northern Australia, the Pacific Islands, China, and Southeast Asia [3]. Jasmine varies in its degree of pubescence, the number of corolla lobes, and the length of the calyx. All these characteristics are often used to differentiate different jasmine species [9]. No clear variation or discontinuity is present because of its wide geographic range. Jasmine is cultivated as *J. bifarium* in the Philippines, *J. amplexicaule* in Pakistan, and *J. aemulum* in Australia [10]. Other species may also be morphologically similar but genetically different from each other. Therefore, it is necessary to identify the diversity in this genus to overcome the ambiguities existing in its classification. The application of different molecular markers is an important preliminary screening tool for diversified genotypes in a natural population [11]. Therefore, different molecular markers, such as random amplification of polymorphic DNA (RAPD) [12,13,14,15,16,17], amplified fragment length polymorphism [18], inter-simple sequence repeats [19,20,21], and simple sequence repeats (SSR) [22], have been used successfully for assessing genetic diversity in *Jasminum*.

Despite the widespread existence of wild *Jasminum* in Pakistan, there are hardly any data regarding their genetic diversity. Some species were not included in previous studies on *Jasminum* genetic diversity. This study sought to use ISSR markers to comprehensively assess the genetic diversity among 28 *Jasminum* accessions that were collected from different localities of Pakistan as shown in Table 1.

## 2. Results

**Figure 1:**
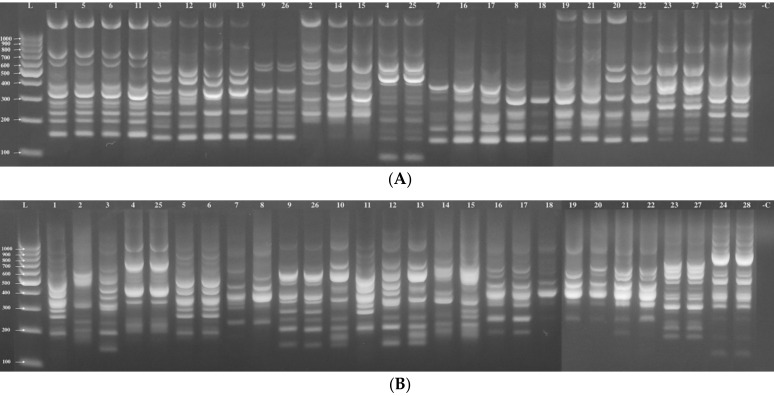

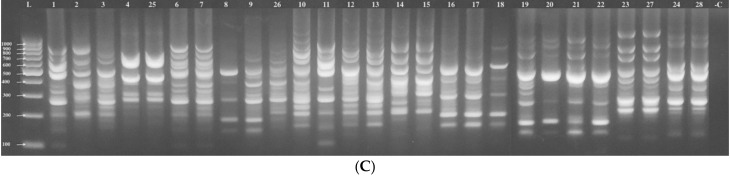
PCR amplification of ISSR primers: primer 15 (**A**), primer 18 (**B**), and primer 21(**C**).

In this study, 21 ISSR primers were used, which produced a total of 570 amplified fragments of different sizes for 28 *Jasminum* accessions, with an average of 27.14 bands per primer. The size of the amplified fragments was 90 to 2200 bp, as shown in Table 2. The ISSR pattern achieved with primers 15, 18, and 21 is illustrated in Figure 1A–C, as an example. The maximum number of fragments (39 bands) was obtained with primer 16, whereas the minimum number was produced by primers 9 and 10 (18 bands each). Of the 570 fragments, 562 were polymorphic, with a mean polymorphic percentage of 98.26%. The number of polymorphic bands showed variation, from 16 (primer 10) to 38 (primers 16 and 20). The maximum resolving power (Rp) was 24.21, which was exhibited by primer 19, while primer 3 displayed a lower Rp value (13.00). The PIC was higher for primer 6 (0.40) and lower for primer 10 (0.28). The marker index (MI) fluctuated from 4.01 for primer 10 to 12.32 for primer 16, as shown in Table 2.

**Figure 2:**
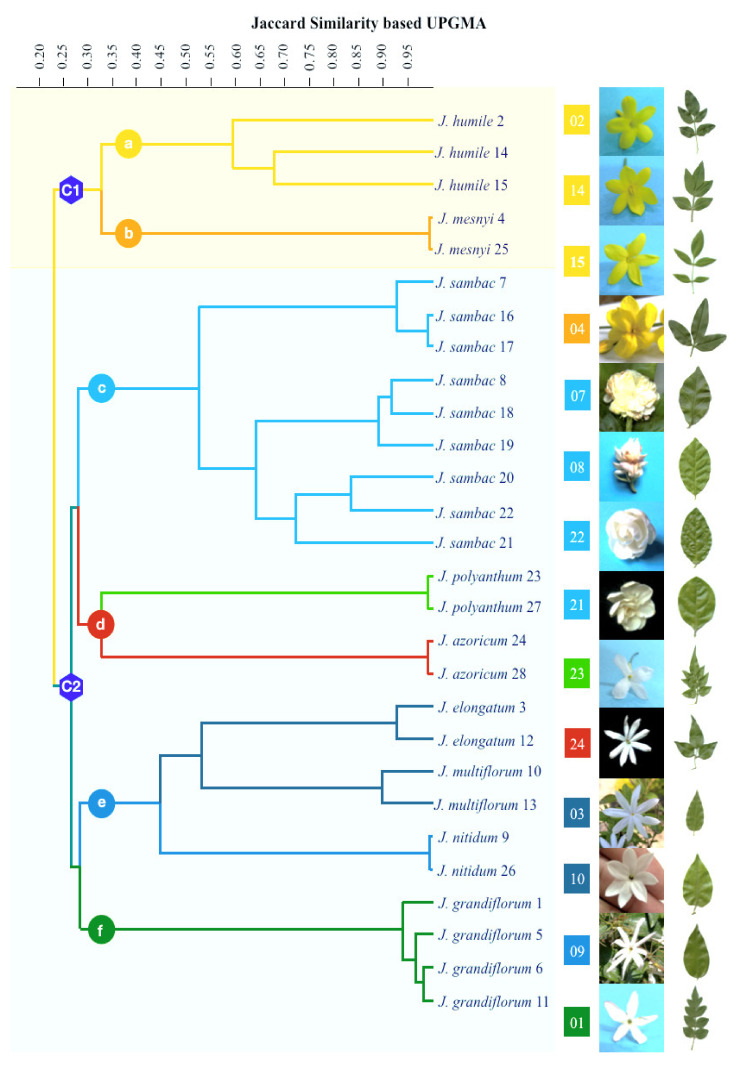
UPGMA dendrogram of the Jaccard similarity matrix representing the genetic relationships among the indicated *Jasminum* accessions.

The ISSR clustering cophenetic coefficient with the Jaccard similarity matrix was 0.992. Consequently, to understand the relationships among species, a similarity matrix was constructed using the 0/1 matrix. The method of the unweighted pair group with a UPGMA arithmetic mean dendrogram based on the Jaccard coefficients separated the 28 accessions into two major clusters, as shown in Figure 2. The first cluster (C1) corresponds to *Jasminum* species with yellow flowers, and the second major cluster (C2) comprises *Jasminum* species with white flowers. C1 was further divided into two subclusters: subcluster a, containing three accessions of *J. humile*, and subcluster b, containing two accessions of *J. mesnyi*. C2, comprising white jasmines, was further divided into four subclusters (subcluster c, with nine accessions of *J. sambac*; subcluster d, with four accessions of *J. polyanthum* and *J. azoricum*; subcluster e, with six accessions of *J. nitidum*, *J. multiflorum*, and *J. elongatum*; and subcluster f, with four accessions of *J. grandiflorum*) with 25–27% similarity among all four subclusters of white jasmines. Among these subclusters, subcluster c was sister to subcluster d, whereas subcluster e was sister to subcluster f. Subcluster c was further clustered into three groups of *J. sambac*: single flower (SF), semi-double flower (SDF), and double flower (DF). The second subcluster of white jasmines, subcluster d, was divided into separate groups each for *J. polyanthum* and *J. azoricum.* The accessions of *J. sambac* and *J. humile* showed intraspecific variations and were separated by a considerable distance, whereas the accessions of all the remaining species were not detached significantly from each other within their species. These intraspecific variants showed more than 90% genetic similarity.

**Figure 3:**
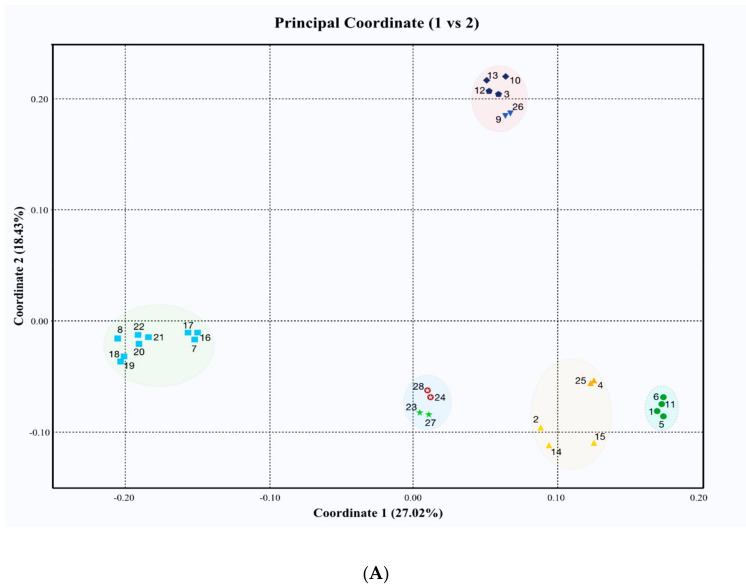

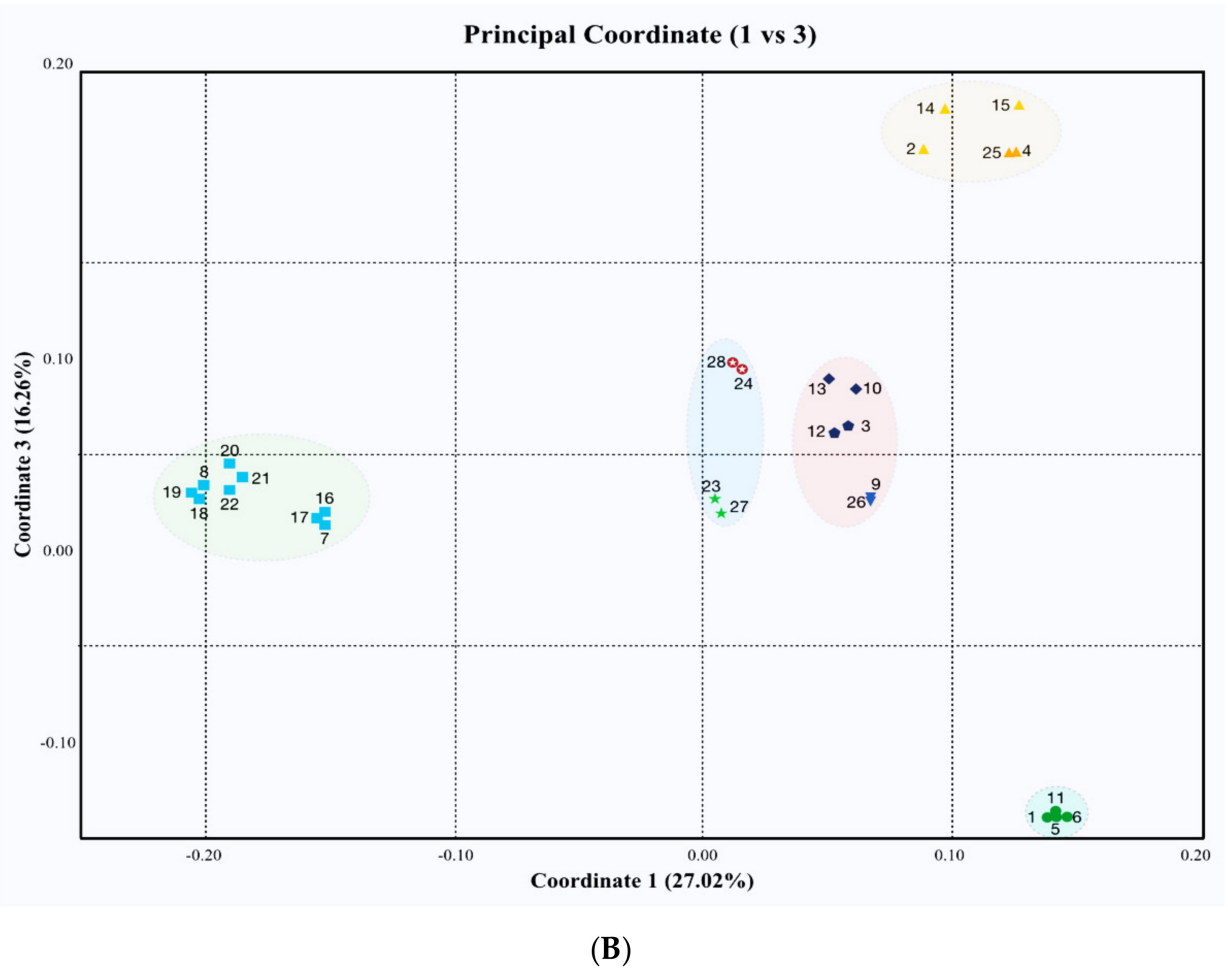
ISSR-data-based PCoA biplot of the genetic distance matrix in the studied species of *Jasminum*: (**A**) (PC1 vs. PC2) and (**B**) (PC1 vs. PC3). The color and shapes represented the species as *J. sambac* (■, blue)*, J. polyanthum* (★, green)*, J. azoricum* (✪, red)*, J. grandiflorum* (●, green)*, J. nitidum* (▼, blue)*, J. elongatum* (⬟, blue)*, J. multiflorum* (♦, blue)*, J. mesnyi* (▲, orange) and *J. humile* (▲, yellow).

In addition, using Gower’s coefficient, PCoA was performed based on the genetic distance matrix to better visualize the genetic diversity of the studied *Jasminum* species. The three principal axes showed 27.02%, 18.43%, and 16.26% of the genetic variance, with a total of 61.71%. The PCoA biplot (Figure 3A) separated three subclusters, c, d, and e. Similarly, the PCoA biplot (Figure 3B) produced clearly separated major clusters, comprising yellow jasmines (subclusters a and b) and a white-flowered subcluster f. Therefore, by using three coordinates in this way, we were able to place all accessions into five distinct groups: four subclusters of white jasmines and one cluster of yellow jasmines (encircled).

Population analysis showed diversity at the species level in only *J. humile* and *J. sambac*. The other species have a small population size, and there was no increase in the number of species defined in this study due to negligible morphological differences among the accessions, with the exception of a few that had considerable variation. The population analysis was performed due to the wider diversity in the floral morphologies of *J. humile* and *J. sambac.* The data regarding these species demonstrate the greatest intraspecific diversity of the studied species based on the highest values for polymorphic percentage, number of effective alleles, Nei’s genetic diversity, Nei’s unbiased diversity, and Shannon’s information index. The lowest values for the above-described population parameters were identified for both *J. mesnyi* and *J. nitidum* (Table 3).

Based on Nei’s unbiased genetic identity and pairwise population matrix at the species level, the highest identity (0.804) was observed between *J. multiflorum* and *J. elongatum*. However, a lower identity (0.575) was noticed between *J. mesnyi* and *J. polyanthum* (Table 4).

The values of PhiPT were statistically significant (*p* = 0.001), indicating additional association based on interspecies variation. The genetic variation in the nine populations recorded was 79% (PhiPT = 0.787), as shown in Table 5.

## 3. Discussion

Given that *Jasminum* is a large and diverse genus in the Oleaceae family, the delimitation of its diversity and species must be carried out carefully. In addition, the usefulness of taxonomy-based classification into different sections needs to be determined. The wide geographic distribution of the genus produces continuous variations in its characteristics, which creates difficulties in the delimitation of genotypes in terms of their intraspecific and interspecific boundaries. *Jasminum* is divided into five sections, *Jasminum*, *Unifoliolata*, *Trifoliolata*, *Primulina*, and *Alternifolia*, based on flower color, leaf shape, and leaf arrangement. Representatives from each section were included in this study to identify their relationships. A diversity analysis of *Jasminum* was also performed on the collected variants using ISSR markers, which showed the intraspecific and interspecific variations and the relationship among the sections. Many studies have validated the use of ISSR markers for genomic characterization in the Oleaceae family [23,24,25,26] and many other plants, such as *Batrachium* [27], *Pistacia* [28], and *Aerva* [29].

In this study, we found higher polymorphism levels in the investigated *Jasminum* accessions, with values of 88.89–100% and a mean of 98.26%, as shown in Table 2. Similarly, Ghehsareh et al. [20] investigated 53 *Jasminum* accessions from Iran using 21 ISSR markers and evaluated their genetic diversity with high polymorphism (90.64%). Yohanan et al. [21] used 10 primers for genetic diversity evaluation within 40 accessions of 23 *Jasminum* species from India, with 100% polymorphism [21]. In addition, Nirmala et al. [18] assessed the genetic similarities among 48 accessions of 26 *Jasminum* species using 10 AFLP markers, of which four displayed 90.5% polymorphism. Qiu et al. [19] used 10 ISSR primers and reported genetic diversity among 30 accessions of *J. sambac*. Li and Zhang [22] used microsatellite markers to analyze *J. sambac* wild germplasm recourses, where six displayed polymorphisms and seven displayed fixed heterozygosity with two alleles. Phithaksilp et al. [17] validated the phylogenetic relationship between 30 genotypes of *Jasminum* with 10 RAPD markers with an overall polymorphism of 71.43%. These studies have shown a high degree of polymorphism with higher levels of genetic diversity in *Jasminum* by using different molecular markers. Furthermore, Raja [30] detected somaclonal variation in *J. auriculatum* using RAPD. Zietkiewicz et al. [31] established ISSR analysis to study diversity among plant species. This method overcomes some of the limitations faced when using other DNA markers. ISSR markers can provide more reliable and reproducible results for polymorphism due to their higher primer lengths and annealing temperatures [32]. The primers used in our study were previously used in other genera of the Oleaceae family, including *Syringa* [33] and *Olea* [26], for which high polymorphism percentages (92.37% and 93.42%) were found.

The values for PIC of the markers were recorded in the range of 0.31–0.40, as shown in Table 2. The PIC depends on the detectable allele number, and its frequency distribution is equal to the gene diversity. The markers that were equally distributed in the population had a higher PIC, which remained up to 0.5 in the dominant markers. The EMR was 14.22–38.00 among the 28 *Jasminum* accessions. Ghehsareh et al. [20] also found EMR values of 25.93–48.08 using the same primers in *Jasminum* accessions from Iran. The higher EMRs show the effectiveness of the marker system for diversity-focused studies [34,35]. The values for marker index (MI) (4.06–12.32) are in accordance with the outcomes of Ghehsareh et al. [20]. The MI is used to calculate the complete effectiveness of marker systems, and a higher value is always associated with better effectiveness of the procedure [34,35,36]. The Rp observed ranged between 13.00 and 24.21. A primer’s ability to differentiate among higher genotype numbers can be represented by the Rp [37,38]. According to Prevost and Wilkinson [36], the Rp is a tool to measure a primer’s capacity to differentiate among accessions. Primers with higher Rp values normally show improved performance in differentiating between genotypes.

The UPGMA dendrogram based on the Jaccard coefficients distributed 28 accessions into two main clusters, as shown in Figure 2. The species placed in the first cluster had yellow flowers with no or less fragrance, while the species in the second cluster had fragrant white flowers. The jasmines with yellow flowers belong to section *Alternifolia*, having alternate leaves, and section *Primulina*, having opposite leaves. The combination of both sections (*Alternifolia* and *Primulina*) showed their close relationship as compared to the white-flower sections (*Unifoliolata, Trifoliolata*, and *Jasminum*), a finding, which was also reported in the molecular phylogenetic investigations of the Oleaceae family by Wallander and Albert [39] and Dupin et al. [40]. This combination might be due to the use of a single species from each section of jasmines with yellow flowers, which may not clearly resolve their relationship. Nevertheless, it is worth mentioning that this section has less similarity (33%) with the section *Alternifolia* because of its opposite leaves, and is similar to jasmines with white flowers. Therefore, section *Primulina* could be predicted to be the point of transition from yellow to white jasmines. This state of transition is also evident from the study of Lee et al. [41], who found evidence of several gene relocation, inversion, deletion, and duplication events in *Jasminum* and *Menodora* and inferred their evolutionary relationship. Rower [42] studied the fruit development stages in *J. mesnyi* and *Menodora* and found that the discontinuity between them is not greater than that between the other sections of *Jasminum*. In another study, Rower [43] found more similarities in many fruit characteristics of *Menodora* with sections *Alternifolia* and *Primulina* compared to other sections of *Jasminum*. Therefore, the grouping of both yellow-flower sections in a single cluster is not unusual as they are the early descendants of this tribe compared with all white-flower jasmines, which evolved independently. Yohanan et al. [21] also proposed that the members of section *Alternifolia* are the ancestors of other *Jasminum* species.

All white-flower jasmines constituted a separate cluster that was further divided into four subclusters, as shown in Figure 2. Subcluster c placed nine accessions of *J. sambac* into three groups: single flower, semi-double flower, and double flower. Subcluster c belongs to section *Unifoliolata*, which has simple, glabrous leaves with fragrant flowers and is sister to multifoliate subcluster d species *J. polyanthum* (section *Jasminum*) and *J. azoricum* (section *Trifoliolata*) but not to *Unifoliolata* species of subcluster e, such as *J. nitidum*, *J. multiflorum*, and *J. elongatum.* These three species of subcluster e have many common characteristics, such as white, star-shaped flowers and opposite, unifoliate leaves with few differences like *J. nitidum* has shiny glabrous lanceolate or ovate leaves, whereas *J. multiflorum* and *J. elongatum* have pubescent ovate–lanceolate leaves with an acuminate apex and broadly ovate leaves with an acuminate apex, respectively. Here, all members of section *Unifoliolata* were divided into two different subclusters (c and e), with the inclusion of *J. azoricum* and *J. polyanthum* (subcluster d), showing that the species of these two *Unifoliolata* subclusters (c and f) are more distantly related. Such combinations have also been reported by Ghehsareh et al. [20] and Yohanan et al. [21]. Subcluster f contained all the *J. grandiflorum* accessions from section *Jasminum*, having pinnate leaves with five to seven leaflets. However, one pinnate-leaf species, *J. polyanthum*, was placed with the trifoliate species *J. azoricum* (subcluster d), confirming that the standard classification based on leaf shape and arrangement is not supported genetically [44]. This could be due to the evolution of leaf morphology (simple or compound) during multiple independent evolutionary events because their genetic makeup is distantly related, as confirmed in our study and many other molecular-based studies [14,20,21].

This study further elucidated the variations between accessions of the same species. For example, for *J. sambac* accessions, higher values for polymorphism percentage, Shannon’s information index, Nei’s genetic diversity, effective allele number, and unbiased Nei’s genetic diversity were found, due to greater variation. The nine accessions of *J. sambac* clearly resolved into three groups: single flower, semi-double flower, and double flower. This grouping of *J. sambac* is in complete agreement with the findings of Ghehsareh et al. for Iranian jasmine species [20]. Similarly, *J. humile* showed the second-most genetic diversity based on greater variation among its accessions. Maximum genetic identities were displayed among *J. elongatum* and *J. multiflorum* due to similarities in most of their characteristics, for example, growth habit, pubescence ovate–lanceolate to broadly ovate leaves with an acuminate apex, and star-shaped flowers with narrowly lanceolate to elliptic corolla lobes. The accessions of other species, namely *J. grandiflorum*, *J. multiflorum*, *J. elongatum*, *J. nitidum*, *J.* *polyanthum*, and *J. azoricum*, did not show the considerable intraspecific variation. All these species showed higher intraspecific similarities among their accessions, which could be due to asexual propagation of cultivated species and a lower number of naturally found wild accessions in this study.

The PCoA based on genetic distance matrix differentiated the species and their variants with better accuracy. These PCoA results correspond highly with the UPGMA clustering and the first three axes explained 61.71% of the genetic variance. In previous studies, distylous morph-specific patterns were observed in *J. fruticans* [45,46] and *J. odoratissimum* [47]. The variation in floral morphology particularly lays the foundation for divergence of the species [45,47,48]. In this study, the accessions of *J. humile* (2, 14, and 15) were far apart from each other and collected from regions where they grow in the wild, and such morph-specific patterns were also observed in these accessions, which could be the reason for their genetic variation.

The analysis of molecular variance (AMOVA) performed for all *Jasminum* accessions showed statistically significant PhiPT values (*p* = 0.001), supporting the interspecific variation that is more likely because of the utilization of different species as populations.

Conclusively, the genetic variations within the genus *Jasminum* was assessed by using the ISSR molecular marker system. The results provide a preliminary indication of genetic diversity among *Jasminum* accessions from Pakistan. The computed results show the close identity between *J. multiflorum* and *J. elongatum*. The outcomes further present an augmented distance between the accessions of *J. humile*, which are characterized by distylous morph-specific pattern. Similarly, the accessions of *J. sambac* were transformed into three groups according to their single-, semi-double-, and double-flowering nature. On a broader spectrum, the white and yellow jasmines were clearly separated, with the indication of character evolution from yellow to white flowering, along with multiple and independent evolution of sections *Unifoliolata* and *Jasminum.* These multiple and independent evolutionary events show that the classification into diverse sections on the basis of leaf morphology, leaf arrangement, and flower color is not supported genetically, whereas these sections are paraphyletic. Therefore, our study provides preliminary information regarding the intraspecific and interspecific diversity in *Jasminum* and a baseline database to assist biologists and taxonomists in species delimitation. The findings will help in phylogenetic-based studies of this genus, particularly in resolving existing ambiguities in its classification.

## 4. Materials and Methods

### 4.1. Sampling of Plants

The experimental work was performed in Prof. Daniel Potters Laboratory, College of Agriculture and Environmental Sciences, UC Davis, USA. Field surveys were conducted in summer, and 28 *Jasminum* accessions, representing nine species (*J. grandiflorum* (4 genotypes), *J. azoricum* (2 genotypes), *J. multiflorum* (2 genotypes), *J. humile* (3 genotypes), *J. elongatum* (2 genotypes), *J. mesnyi* Hance. (2 genotypes), *J. nitidum* (2 genotypes), *J. polyanthum* (2 genotypes), and *J. sambac* (9 genotypes; 3 semi-double-flower, 3 double-flower, and 3 single-flower types)) (Table 1) were collected from diverse regions of Pakistan. The wild and cultivated *Jasminum* accession germplasm was established in the Horticulture Department, Pir Mehr Ali Shah Arid Agriculture University, Rawalpindi, Pakistan. The sample collection details, along with GPS coordinates, are given in Table 1.

### 4.2. Plant Identification and Herbarium Preparation

Plants were primarily identified by botanical experts, and their herbarium specimens were prepared. These specimens were submitted to the Pakistan Museum of Natural History (PMNH), Islamabad, Pakistan, as herbarium specimen vouchers (Table 1).

### 4.3. Total Genomic DNA Extraction

Dried leaf samples were taken from the herbarium specimens and washed with distilled water, followed by 70% ethanol, and then dried with silica gel. Genomic DNA was extracted by grinding the dried leaf samples of 28 accessions into a fine powder. The DNeasy Plant Mini Kit (Qiagen) was used for DNA extraction following the prescribed protocols.

### 4.4. Quantification of Genomic DNA

After extraction, the quality and quantity of the extracted DNA were evaluated using an ND 2000 spectrometer (NanoDrop Technologies, Wilmington, USA) by assessing the absorbance at A260 and A280 [49]. The extracted genomic DNA was diluted to a concentration of 15 ng/μL for the ISSR reaction using standard methods [20].

### 4.5. ISSR Primers and Their PCR Amplification

In this study, 21 ISSR primers (Table 2) were selected that had shown successful results in previous studies on three Oleaceae genera: *Syringa* [33], *Jasminum* [20,21], and *Olea* [25]. The inter-repeat region of the *Jasminum* accession genome was amplified with ISSR primers following standard PCR procedures, as described by Ghehsareh et al. [20].

Briefly, the PCR profile for the amplification of genomic regions using an ISSR marker is given in Table 2. Each 25 μL of the PCR reaction mixture contained 0.5 μL of dinucleotide triphosphate (2.5 mM each; total 10 mM), 0.7 μL of MgCl_2_ (50 mM), 17.55 μL of double-distilled water, 2.5 μL of 10× PCR buffer, 0.25 μL (5 U/μL) of Taq-polymerase (Qiagen, USA), 1.5 μL (50 μM) of primer, and 2 μL of template DNA (15 ng/μL). A sample with no DNA was taken as a negative control. Gel electrophoresis was carried out to visualize the amplified genomic region in a gel using DNA standard size markers of 1 kb (N-3232L, Biolabs Company), as described in Ghehsareh et al. [20].

### 4.6. Data Analysis

All the amplified fragments were deliberated as a dominant locus-specific allele. The bands obtained from each locus were noted as absent (0) or present (1), and the ISSR loci data matrix was gathered for more scrutiny. The UPGMA dendrogram based on the arithmetic mean unweighted pair group and the similarity matrices were produced with the Jaccard coefficient. The coefficient of cophenetic correlation was calculated to assess the goodness of fit for the dendrogram and the similarity matrices with a test, as described by Mantel [50]. Principal coordinate analysis (PCoA) was performed with Gower’s coefficient using Paleontological Statistics (PAST) software, version 3 [51]. To distinguish among accessions, the capacity of each primer studied was evaluated on the basis of the marker index (MI) [34], polymorphic information content (PIC) [52], and resolving power (Rp) [38]. The PIC was estimated by the following formula:

(1) $$\mathrm{PIC}=1-{pi}^{2}-{qi}^{2}$$

where q is the null allele frequency and p is the visual allele frequency [53].

In addition, MI = PIC × polymorphic loci number and Rp = ΣIb, where b is the formativeness band, taking values 1 − (2 × (0.5 − p)), and p is the amount of each genotype encompassing a band.

To calculate the polymorphic loci percentage, GenAlEx 6.503 was used [54]. The MI, PIC, Rp, and effective multiplex ratio (EMR) were premeditated with Microsoft Excel, version 16.9. Diversity was determined based on calculation of Nei’s index and Shannon’s information index (I) [55]. AMOVA was used to estimate the variance between populations. Wright’s F_ST_ analogue is considered a PhiPT value, and assessment of PhiPT with AMOVA provides species variation information. GenAlEx 6.503 was used to quantify the pairwise genetic distance with Nei’s unbiased genetic distance among populations, Shannon’s information index (I), genetic diversity index (He), different allele number (Na), polymorphic loci percentage, effective allele number (Ne), and ANOVA.

## Figures and Tables

**Table 1:** Sampling details of *Jasminum* species obtained from different regions of Pakistan.

*Jasminum* spp.	Type	No. of Genotypes	Acc. No.	Taxon ID	Locality	Latitude	Longitude	Altitude (ft.)
*J. grandiflorum* L.		4	1	044522	Islamabad	33°44′02″	73°02′10″	2026
			5	044526	Rawalpindi	33°55′23″	73°23′25″	5784
			6	044527	Abbottabad	34°14′01″	73°17′53″	6585
			11	044533	Peshawar	34°01′06″	71°34′30″	1056
*J. elongatum* Willd.		2	3	044524	Rawalpindi	33°38′46″	73°04′50″	1680
			12	044534	Islamabad	33°39′49″	73°03′47″	1691
*J. multiflorum* (Burm. F) Andrews		2	10	044532	Haripur	33°59′40″	72°56′02″	1752
			13	044535	Kotli AJK	33°26′12″	74°05′12″	3419
*J. humile* L.		3	2	044523	Islamabad	33°46′26″	73°05′16″	3976
			14	044536	Mansehra	34°21′16″	73°10′37″	4194
			15	044537	Mansehra	34°05′56″	73°20′56″	6147
*J. mesnyi* Hance		2	4	044525	Islamabad	33°41′28″	73°04′20″	1828
			25	044547	Islamabad	33°43′56″	73°02′05″	2014
*J. nitidum* Skan.		2	9	044530	Rawalpindi	33°38′45″	73°04′51″	1680
			26	044548	Kasur	30°59′40″	73°48′55″	641
*J. sambac* L.	Double flower	3/9	7	044528	Sargodha	32°04′20″	72°40′46″	631
			16	044538	Kasur	30°59′23″	73°48′24″	642
			17	044539	Lahore	31°33′13″	74°19′47″	727
	Semi-double flower	3/9	8	044529	Sargodha	32°04′20″	72°40′46″	631
			18	044540	Lahore	31°33′18″	74°19′45″	725
			19	044541	Rawalpindi	33°38′45″	73°04′51″	1681
	Single flower	3/9	20	044542	Rawalpindi	33°38′45″	73°04′51″	1681
			21	044543	Jhelum	32°56′24″	73°24′11″	980
			22	044544	Rawalpindi	33°38′45″	73°04′51″	1681
*J. polyanthum* Franch		2	23	044545	Rawalpindi	33°38′45″	73°04′51″	1681
			27	044549	Kasur	30°59′40″	73°48′55″	641
*J. azoricum* L.		2	24	044546	Rawalpindi	33°38′45″	73°04′52″	1681
			28	044550	Karachi	24°52′20″	67°04′43″	136

**Table 2:** ISSR primer data among 28 accessions of *Jasminum* species.

Primer No.	Primer Sequence (5’-3’)	Annealing Temp. (°C)	Size of Fragment Range (bp)	Band Number Per Primer (*n*)	Polymorphic Loci Number (np)	Pp (%)	EMR	PIC	MI	Rp
1	(CA)8G	49	120–1100	24	24	100.0	24.00	0.36	8.70	17.93
2	(GA)8YCa	52.7	100–1700	23	23	100.0	23.00	0.32	7.38	14.71
3	(TC)8G	52	210–2200	27	27	100.0	27.00	0.33	8.80	13.00
4	(AC)8GG	55.6	135–2100	30	30	100.0	30.00	0.36	10.90	17.29
5	(AC)8GA	45.8	120–1500	26	26	100.0	26.00	0.33	8.50	19.86
6	(GT)8YC	50.5	150–1500	29	29	100.0	29.00	0.40	11.57	19.50
7	(AG)8GC	54.9	135–1250	26	25	96.15	24.04	0.34	8.15	17.64
8	(AC)8YA	46.5	175–1150	24	23	95.83	22.04	0.35	7.61	16.79
9	(TG)8C	51.7	180–700	18	18	100.0	18.00	0.37	6.67	15.14
10	(AG)8YG	52.5	195–1300	18	16	88.89	14.22	0.28	4.01	15.07
11	(GGAGA)3GT	56.2	160–1500	34	34	100.0	34.00	0.35	11.79	21.57
12	(GA)8T	46.5	205–1400	21	20	95.24	19.05	0.39	7.51	17.00
13	(AG)8T	46.5	160–1200	29	29	100.0	29.00	0.35	10.20	21.29
14	(GT)8YA	48.7	180–1200	20	18	90.00	16.20	0.34	5.54	17.36
15	(AG)8YT	48.7	90–1400	24	24	100.0	24.00	0.31	7.44	17.29
16	(GA)8C	52.3	150–2000	39	38	97.44	37.03	0.33	12.32	23.43
17	(TC)8C	52.2	160–1200	24	24	100.0	24.00	0.32	7.65	15.43
18	(CA)9GT	48.7	120–1300	31	31	100.0	31.00	0.35	10.82	18.36
19	(GA)9T	46.8	90–850	35	35	100.0	35.00	0.33	11.60	24.21
20	(GA)9A	47	150–1550	38	38	100.0	38.00	0.31	11.87	17.14
21	(GA)9C	52.3	100–1200	30	30	100.0	30.00	0.35	10.58	22.78
			TOTAL	570	562	2063.55	-	-	-	-
			MEAN	27.14	26.76	98.26	-	-	-	-

Pp (polymorphic percentage); PIC (polymorphic information content) = 1 − pi^2^ − qi^2^, where q is the null allele frequency and p is the visual allele frequency. EMR (effective multiplex ratio) = np (np/*n*), where *n* is the total loci number and np is the polymorphic loci number; Rp (resolving power) = ΣIb, where Ib = 1 − (2 × |0.5 − p|); MI (marker index) = PIC × EMR.

**Table 3:** Polymorphic loci percentage, sample size, effective allele number, different allele number, Shannon’s information index, and Nei’s genetic diversity of the studied *Jasminum* populations (SE and mean over the loci for each population).

Pop.	N	Na	Ne	I	He	uHe	P%
*J. grandiflorum*	4	0.414 (0.023)	1.017 (0.005)	0.016 (0.004)	0.010 (0.003)	0.012 (0.003)	3.16%
*J. elongatum*	2	0.368 (0.022)	1.017 (0.005)	0.015 (0.004)	0.010 (0.003)	0.014 (0.004)	2.46%
*J. multiflorum*	2	0.414 (0.024)	1.027 (0.006)	0.023 (0.005)	0.016 (0.003)	0.021 (0.004)	3.86%
*J. sambac*	9	0.830 (0.038)	1.208 (0.014)	0.179 (0.011)	0.120 (0.008)	0.127 (0.008)	33.16%
*J. humile*	3	0.656 (0.034)	1.151 (0.013)	0.128 (0.010)	0.087 (0.007)	0.104 (0.008)	22.28%
*J. mesnyi*	2	0.305 (0.019)	1.001 (0.001)	0.001 (0.001)	0.001 (0.001)	0.001 (0.001)	0.18%
*J. nitidum*	2	0.339 (0.020)	1.001 (0.001)	0.001 (0.001)	0.001 (0.001)	0.001 (0.001)	0.18%
*J. polyanthum*	2	0.361 (0.020)	1.002 (0.002)	0.002 (0.001)	0.001 (0.001)	0.002 (0.001)	0.35%
*J. azoricum*	2	0.351 (0.020)	1.002 (0.002)	0.002 (0.001)	0.001 (0.001)	0.002 (0.001)	0.35%
Grand mean	3.111	0.449 (0.009)	1.047 (0.003)	0.041 (0.002)	0.027 (0.001)	0.031 (0.002)	7.33% (3.99%)

Na (different allele number); Ne (effective allele number) = 1/(p^2^ + q^2^); I (Shannon’s information index) = −1 × (p × Ln (p) + q × Ln(q)); He (heterozygosity expected) = 2pq; uHe (expected heterozygosity unbiased) = (2N/(2N − 1))He where, by assuming diploid binary data and Hardy–Weinberg equilibrium, q = (1 − Band Freq.)^0.5^ and *p* = 1 − q; P (percentage of polymorphic loci).

**Table 4:** A pairwise population matrix of Nei’s unbiased genetic identity.

	*J. gf*	*J. el*	*J. mt*	*J. sb*	*J. hu*	*J. ms*	*J. nt*	*J. po*	*J. az*
*J. gf*	1.000								
*J. el*	0.616	1.000							
*J. mt*	0.601	0.804	1.000						
*J. sb*	0.654	0.706	0.690	1.000					
*J. hu*	0.649	0.657	0.645	0.724	1.000				
*J. ms*	0.603	0.597	0.611	0.648	0.734	1.000			
*J. nt*	0.626	0.759	0.736	0.684	0.632	0.605	1.000		
*J. po*	0.616	0.601	0.578	0.687	0.644	0.575	0.605	1.000	
*J. az*	0.586	0.604	0.604	0.672	0.643	0.582	0.566	0.642	1.000

*J. gf* (*Jasminum grandiflorum*); *J. el* (*Jasminum elongatum*); *J. mt* (*Jasminum multiflorum*); *J. sb* (*Jasminum sambac*); *J. hu* (*Jasminum humile*); *J. ms* (*Jasminum mesnyi*); *J. nt* (*Jasminum nitidum*); *J. po* (*Jasminum polyanthum*); *J. az* (*Jasminum azoricum*).

**Table 5:** AMOVA summary for *Jasminum* accessions.

Source	df	SS	MS	Est. var.	%
Among pop.	8	2267.313	283.414	88.829	79%
Within pop.	19	457.222	24.064	24.064	21%
Total	27	2724.536		112.894	100%
PhiPT	0.787 P (rand ≥ data) 0.001				

Probability, P (rand >= data), for PhiPT is on the basis of the standard permutation across the complete data set; PhiPT = AP/(WP + AP) = AP/TOT, where WP is the estimated variance within populations and AP is the estimated variance among populations.
